# Enhanced recovery after surgery (ERAS) protocol improves patient outcomes in free flap surgery for head and neck cancer

**DOI:** 10.1007/s00405-023-08292-3

**Published:** 2023-11-08

**Authors:** Teija Nieminen, Laura Tapiovaara, Leif Bäck, Andrew Lindford, Patrik Lassus, Lasse Lehtonen, Antti Mäkitie, Harri Keski-Säntti

**Affiliations:** 1 Department of Perioperative and Intensive Care Medicine, University of Helsinki and Helsinki University Hospital, Haartmaninkatu 4, PO Box 340, 00029 HUS Helsinki, Finland; 2 Department of Otorhinolaryngology, Head and Neck Surgery, University of Helsinki and Helsinki University Hospital, Helsinki, Finland; 3 Department of Plastic Surgery, University of Helsinki and Helsinki University Hospital, Helsinki, Finland; 4 HUS Diagnostic Center, University of Helsinki and Helsinki University Hospital, Helsinki, Finland; 5 Research Program in Systems Oncology, Faculty of Medicine, University of Helsinki, Helsinki, Finland

**Keywords:** ERAS, Enhanced recovery after surgery, Head and neck cancer, Free flap surgery, Microvascular reconstruction

## Abstract

**Background:**

In recent years, enhanced recovery after surgery (ERAS) guidelines have been developed to optimize pre-, intra-, and postoperative care of surgical oncology patients. The aim of this study was to compare management outcome of patients undergoing head and neck cancer (HNC**)** surgery with free flap reconstruction at our institution before and after the implementation of the ERAS guidelines.

**Methods:**

This retrospective study comprised 283 patients undergoing HNC surgery with free flap reconstruction between 2013 and 2020. Patients operated before and after the implementation of the ERAS protocol in October 2017 formed the pre-ERAS group (*n* = 169), and ERAS group (*n* = 114), respectively.

**Results:**

In the pre-ERAS group the mean length of stay (LOS) and intensive care unit length of the stay (ICU–LOS) were 20 days (range 7–79) and 6 days (range 1–32), and in the ERAS group 13 days (range 3–70) and 5 days (range 1–24), respectively. Both LOS (*p* < 0.001) and ICU–LOS (*p* = 0.042) were significantly reduced in the ERAS group compared to the pre-ERAS group. There were significantly fewer medical complications in the ERAS group (*p* < 0.003). No difference was found between the study groups in the surgical complication rate or in the 30-day or 6-month mortality rate after surgery.

**Conclusions:**

We found reduced LOS, ICU–LOS, and medical complication rate, but no effect on the surgical complication rate after implementation of the ERAS guidelines, which supports their use in major HNC surgery.

**Supplementary Information:**

The online version contains supplementary material available at 10.1007/s00405-023-08292-3.

## Introduction

The idea behind the current Enhanced Recovery After Surgery (ERAS) protocols were first introduced by Kehlet [1]. The aim is to optimize recovery from major surgery through patient guidance and multidisciplinary collaboration, taking into account evidence-based perioperative care [2]. This treatment model was originally created for patients undergoing colorectal surgery [3]. The ERAS Society was officially registered in 2010, and since then, ERAS guidelines have been applied to many surgical fields. For major head and neck surgery with free flap reconstruction, the recommendations for optimal perioperative care were published by the ERAS Society in 2017, to optimize clinical outcome by influencing perioperative care in an evidence-based and structured manner [4].

Reconstruction of tissue defects with microvascular  free flaps is the standard of care in the surgical treatment of advanced head and neck cancer (HNC). However, complications tend to be common, reportedly even in over 70% of patients [5]. Many of these patients are older, with significant comorbidities, and are often heavy users of alcohol and tobacco predisposing them to both surgical and medical complications.

Several studies on HNC patients undergoing free tissue reconstruction have compared outcome before and after implementation of ERAS guidelines (Supplementary Table 1). The most common finding seems to be significantly reduced hospital length of stay (LOS). In a systematic review and meta-analysis recently published by Chorath et al., the number of wound complications was lower, LOS shorter, and the readmission rate reduced in patients treated using ERAS guidelines [6].

The aim of this study was to investigate the effects of the implementation of ERAS protocol at our tertiary care center. Special emphasis was placed on the effects on LOS, length of intensive care stay (ICU–LOS), complications, and mortality within 6 months after operation. We hypothesized that ERAS implementation would reduce these parameters.

## Materials and methods

Retrospective data were collected from the hospital registry at the Helsinki University Hospital (HUS) (Helsinki, Finland) and comprised 283 patients undergoing HNC surgery with free flap reconstruction between 2013 and 2020. The ERAS protocol was implemented in October 2017. Patients operated before that timepoint comprised the pre-ERAS group (*N* = 169). Patients operated after October 2017 comprised the ERAS group (*N* = 114). The collected parameters to be compared between the groups were patient demographics (age, sex), ACE-27 comorbidity index [7], tumor location, TNM classification and stage, the type of free flap used, medical and surgical complications, mortality within 30 days or 6 months after surgery, LOS, ICU–LOS, and the need for re-operation or re-admission to intensive care. The primary endpoints were LOS, ICU–LOS, and complications (both surgical and medical).

The ERAS protocol used at our institution was designed by a multidisciplinary group, including otolaryngologist—head and neck surgeons, plastic surgeons, oral and maxillofacial surgeons and anesthesiologists. The cornerstone of our protocol was the recommendation for optimal perioperative care in major HNC surgery with free flap reconstruction published by the ERAS Society, which was modified to match our local operational environment [4]. During the study period, our ERAS protocol was updated once: in October 2018 a recommendation of rapid awakening after operation was added. The principles of our ERAS guidelines are presented in Supplementary Table 2.

The aim of the statistical modelling was to study difference between pre-ERAS and ERAS periods. All data were manually extracted from the electronic hospital charts and transferred to SPSS statistical software. Statistical analyses were performed using IBM SPSS Statistics 25 for Windows (IBM Corp., Armonk, NY) and R language [8].

We modeled continuous variables with linear regression. Three models were applied: Model 1 only with ERAS period as predictor; Model 2 with ERAS period, sex, and age; Model 3 with ERAS period, sex, age, stage, and ACE-27 score. Linear models estimate difference between pre-ERAS and ERAS with 95% confidence intervals.

Dichotomic (no/yes) variables were modelled with modified Poisson regression that estimates relative risk between pre-ERAS and ERAS [9]. This modelling approach was used instead of standard logistic regression, because some of the depended variables have high prevalence, and thus odds ratios produced by logistic regression do not estimate relative risk  so well [10]. Results of modified Poisson regression are presented as relative risks with 95% confidence intervals.

Institutional permission to conduct this study was granted by the Research Administration of the HUS District (HUS/419/2018, HUS/307/2019). This research involved only patient charts, and therefore, no formal Research Ethics Board approval or informed consent was needed according to the Finnish legislation.

## Results

The patient characteristics are shown in Table 1.

**Table 1:** Descriptive statistics of the study cohort

	Pre-ERAS (*n* = 169)	ERAS (*n* = 114)	*p* value
Age, mean (range)	62 (24–86)	63 (23–90)	0.378
Male, *n* (%)	121 (71.6)	79 (69.3)	0.777
ACE-27 score, *n* (%)			0.754
0	49 (29.0)	28 (24.6)	
1	61 (36.1)	47 (41.2)	
2	42 (24.9)	26 (22.8)	
3	17 (10.1)	13 (11.4)	
Tumour site, *n* (%)			
Oral cavity	82 (48.5)	47 (41.2)	
Pharynx	53 (31.4)	36 (31.6)	
Larynx	12 (7.1)	8 (7.0)	
Sinonasal	13 (7.7)	11 (9.6)	
Salivary gland	1 (0.6)	1 (0.9)	
Skin	1 (0.6)	4 (3.5)	
Other	7 (4.1)	7 (6.1)	
Cancer stage, *n* (%)			< 0.001
Stage I	17 (10.8)	3 (3.3)	
Stage II	27 (17.1)	26 (28.3)	
Stage III	20 (12.7)	18 (19.6)	
Stage IVA	87 (55.1)	33 (35.9)	
Stage IVB	6 (3.8)	11 (12.0)	
Stage IVC	1 (0.6)	1 (1.1)	
Free flap used, *n* (%)			
Anterolateral thigh	83 (49.1)	76 (66.7)	
Radial forearm	34 (20.1)	13 (11.4)	
Latissimus dorsi	22 (13.0)	7 (6.1)	
TAPAS	6 (3.6)	5 (4.4)	
Rectus abdominis	2 (1.2)	1 (0.9)	
Scapula	7 (4.2)	3 (2.6)	
Fibula	5 (3.0)	0 (0)	
TDAP	0 (0)	1 (0.9)	
Other	15 (8.9)	8 (7.0)	

ACE-27, Adult Comorbidity Evaluation 27; TAPAS, temporal  artery-based posterior auricular skin flap; TDAP, thoracodorsal artery perforator

In the pre-ERAS and ERAS groups the mean LOS and ICU–LOS were 20 days (range 7–79) and 13 days (range 3–70), and 6 days (range 1–32) and 5 days (range 1–24), respectively. Both LOS and ICU–LOS were statistically reduced by a linear logistic regression model in the ERAS group compared to the pre-ERAS group (Table 2).

**Table 2:** Linear regression model for LOS and ICU–LOS compared to ERAS and pre-ERAS groups

	Adjusted difference (95% CI)	*p* value
Model 1 variables: ERAS		
ICU–LOS	− 1.09 (− 2.13 to − 0.05)	0.04
LOS	− 6.89 (− 4.11 to − 9.67)	< 0.001
Model 2 variables: ERAS, age, sex		
ICU–LOS	− 1.15 (− 0.11 to − 2.18)	0.03
LOS	− 6.83 (− 4.04 to − 9.62)	< 0.001
Model 3 variables: ERAS, age, sex, stage, ACE-27 score		
ICU–LOS	− 0.93 (0.19 to − 2.05)	0.10
LOS	− 6.59 (− 3.47 to − 9.71)	< 0.001

CI, confidence interval; ICU–LOS, intensive care unit-length of stay; LOS, length of stay; ACE-27 score, Adult Comorbidity Evaluation 27

No difference was found in the overall complication rate: in the pre-ERAS and ERAS groups, 115 patients (68%) and 76 patients (67%) had complications (*p* = 0.808), respectively (Table 3). Neither were there any significant differences in the rate of surgical complications between the two groups. The rate of medical complications was significantly lower in the ERAS group using modified Poisson regression model (Table 4). In both the pre-ERAS and ERAS groups, the majority of complications belonged to Clavien–Dindo grades IIIb (pre-ERAS 26.6%, ERAS 21.1%) and IVa (pre-ERAS 26%, ERAS 22.8%) (data not shown) [11].

**Table 3:** Comparison of postoperative data

	Pre-ERAS (*n* = 169)	ERAS (*n* = 114)	*p* value
Mean length of stay, days (range)	20 (7–79)	13 (3–70)	< 0.001
Mean ICU length of stay, days (range)	6 (1–32)	5 (1–24)	0.042
Complications, *n* (%)			
Overall	115 (68.0)	76 (66.7)	0.808
Surgical complication	80 (47.3)	55 (48.2)	0.881
Medical complication	77 (45.6)	32 (28.1)	0.003
One complication per patient	50 (29.6)	55 (48.2)	
Two complications per patient	26 (15.4)	19 (16.7)	
Three or more complications per patient	39 (23.1)	2 (1.8)	
Type of surgical complication, *n* (%)			
Total flap loss	10 (5.9)	14 (12.3)	
Partial flap loss	10 (5.9)	9 (7.9)	
Surgical site hematoma	14 (8.3)	4 (3.5)	
Surgical site infection	23 (13.6)	5 (4.4)	
Fistula	15 (8.9)	9 (7.9)	
Tissue necrosis	26 (15.4)	4 (3.5)	
Other	17 (10.1)	14 (12.3)	
Type of medical complication, *n* (%)			
Pneumonia	20 (11.8)	6 (5.3)	
Sepsis	1 (0.6)	1 (0.9)	
Delirium	33 (19.5)	14 (12.3)	
Arrhythmia	5 (3.0)	2 (1.8)	
Atelectasis/pulmonary edema	11 (6.5)	0 (0)	
Pulmonary embolism	2 (1.2)	1 (0.9)	
Prolonged mechanical ventilation	11 (6.5)	4 (3.5)	
Hypotension/hemodynamic instability	0 (0)	2 (1.8)	
Other	20 (11.8)	7 (6.1)	
Re-operation within 30 days, *n* (%)			
Number of patients	64 (37.9)	35 (30.7)	0.266
Re-admission to ICU/prolonged stay in ICU, *n* (%)	32 (18.9)	43 (37.7)	< 0.001
Died within 30 days, *n* (%)	3 (1.8)	5 (4.4)	0.350
Died within 6 months, *n* (%)	21 (12.4)	14 (12.3)	1.000

SD, standard deviation; ICU, intensive care unit

**Table 4:** Modified Poisson regression model for independent variables comparing ERAS group to pre-ERAS group

	Model 1. (variable: ERAS)	Model 2. (variables: ERAS, age, sex)	Model 3. (variables: ERAS, age, sex, stage, ACE-27 score)
Independent variable	RR (95% CI)	RR (95% CI)	RR (95% CI)
Overall complications	0.96 (0.83–1.15)	0.97 (0.82–1.15)	0.98 (0.83–1.17)
Surgical complications	0.99 (0.78–1.27)	1.00 (0.78–1.28)	1.01 (0.78–1.33)
Medical complications	0.64 (0.46–0.89)*	0.62 (0.45–0.86)*	0.66 (0.47–0.93)*
Type of surgical complication			
Total flap loss	3.19 (1.25–8.15)	3.23 (1.28–8.13)	3.90 (1.42–10.71)
Surgical site hematoma	0.32 (0.09–1.07)*	0.32 (0.10–1.09)*	0.27 (0.06–1.27)*
Surgical site infection	0.61 (0.22–1.70)*	0.64 (0.24–1.72)*	0.54 (0.14–2.07)*
Type of medical complication			
Pneumonia	0.64 (0.25–1.62)*	0.61 (0.24–1.57)*	0.66 (0.23–1.84)*
Delirium	0.60 (0.33–1.10)*	0.58 (0.32–1.05)*	0.62 (0.33–1.14)*
Re-operation within 30 days	0.81 (0.58–1.14)	0.81 (0.58–1.14)	0.90 (0.63–1.28)
Re-admission to ICU/prolonged stay in ICU	1.99 (1.35–2.95)	1.94 (1.32–2.86)	2.02 (1.30–3.15)
Died within 30 days	2.47 (0.60–10.14)	2.27 (0.56–9.23)	1.77 (0.31–10.13)
Died within 6 months	0.99 (0.53–1.86)	0.99 (0.52–1.89)	0.74 (0.30–1.82)

RR, relative risk; CI, confidence interval; ACE-27 score, Adult Comorbidity Evaluation 27; ICU, intensive care unit

*Statistically significant

No statistically significant differences in mortality were found: in the pre-ERAS group the 30-day and 6-month mortality were 1.8% and 12.4%, and in the ERAS group 4.4% and 12.3%, respectively (Tables 3, 4).

The number of re-operations within 30 days in the pre-ERAS and ERAS groups were 64 (37.9%) and 35 (30.7%), respectively (*p* = 0.266) (Table 3).

The rate of re-admissions to the ICU or prolonged stay in the ICU was significantly higher in the ERAS group compared to the pre-ERAS group (38% vs. 19%, *p* < 0.001) (Table 3).

## Discussion

In 2017 at our institution, an evidence-based ERAS recommendation was implemented aiming at improving the quality of perioperative care of patients undergoing major HNC surgery. In this study, we focused on the effects of ERAS guidelines especially on LOS, ICU–LOS, and complication rate. In patients undergoing surgery after implementation of ERAS guidelines, the mean LOS was reduced by 7 days and ICU–LOS by 1 day, but no difference was found in overall or surgical complication rates. However, the rate of medical complications decreased. Our results are in line with several former studies. In most published studies, the LOS has been reduced after implementation of ERAS guidelines, but only in  one study was the complication rate affected (Supplementary Table 1) [5, 12–20].

Postoperative complications are common in HNC surgery with free flap reconstruction and have been associated with increased LOS, morbidity, and decreased overall survival [21]. In our study, the overall complication rate in the pre-ERAS group was 68.0% and in the ERAS group 66.7%. These figures are relatively high compared to some previous studies, in which complication rates of 20.3–41% have been reported [22, 23]. On the other hand, several studies are in line with our study showing higher complication rates of 54–72% [5, 24–26]. However, there may be methodological differences in reporting complications between different studies. In many studies the complication rate has not been significantly altered after implementation of the ERAS protocol [5, 13, 14, 16, 18–20]. In the study by Kiong et al., as in our study, the use of ERAS guidelines resulted in significantly fewer medical complications [17]. In our study the use of the ERAS protocol reduced the rate of pneumonia, pulmonary edema, prolonged mechanical ventilation, delirium, and surgical site infection. We evaluated all complications extremely carefully and might have reported minor complications more precisely than some other studies. However, the high number of complications did not negatively affect the LOS or ICU–LOS, which were comparable to that reported by others. Twomey et al. found that patient mobilization later than 24 h after surgery is associated with all types of complications, including major complications, encouraging early mobilization [27].

The flap loss rate in the ERAS group was surprisingly high (Tables 3, 4). It seems unlikely that the changes in our treatment protocol related to ERAS would affect flap survival. As the recent review by Ronen et al. suggests, around half of surgical complications are preventable, so we will evaluate our flap loss rate carefully in the future [28]. In addition, the number of re-operations was high in both groups compared to other studies [16, 17, 19]. Methodological differences in reporting complications may at least partly explain this finding as we recorded all minor wound revisions as re-operations.

The rate of re-admissions to the ICU or prolonged stay in the ICU was significantly higher in the ERAS group compared to the pre-ERAS group (Table 4). In both groups, the reasons for re-admissions included both re-operations and medical complications. This is of cause contradictory to the aims of using the ERAS guidelines. One explanation could be the increased need for new reconstruction procedures due to increased number of flap losses in the ERAS group. This issue warrants careful preoperative patient assessment and selection.

A major change in our practice along with the implementation of the ERAS protocol has been in the preoperative nutritional care. All patients will be provided with preoperative supplementary nutrition preparations (immunonutrition, Oral Impact^®^) for 1 week before surgery. Mueller et al. showed benefit of preoperative immunonutrition in HNC surgery, i.e., a decrease in the overall complication rate and reduced LOS [29]. Dort et al. recommended that preoperative fasting should be minimized [4]. According to our ERAS protocol, patients are offered a carbohydrate-rich supplement (200 ml) 2 h before anesthesia, which has been shown to reduce postoperative insulin resistance and LOS [30, 31].

In the ERAS guidelines published by the ERAS Society in 2017, and also in our ERAS protocol, antibiotic prophylaxis is recommended to be continued for only 24 h postoperatively [4]. Before we launched the ERAS guidelines, prophylactic antibiotics were used for significantly longer periods at our institution. According to the present results, the number of infections did not increase after the significant shortening of the prophylactic antibiotic treatment. Instead, the rate of surgical site infections and pneumonias decreased, and the changes were statistically significant in the modified Poisson regression model (Table 4). Numerous studies have shown that long-term postoperative antibiotics do not protect against surgical site infections [32–34]. On the contrary, they may cause antibiotic-related complications, such as Clostridium difficile infections [35].

At our institution, prolonged mechanical ventilation after HNC surgery with free flap reconstruction has been routine practice. The patients have been sedated and ventilated overnight in the ICU and extubated or weaned from the ventilator on the day after surgery. However, several ERAS guidelines recommend avoiding prolonged sedation and mechanical ventilation [36–38]. In a study by Clemens et al. rapid awakening protocol in patients undergoing HNC surgery significantly decreased complication rate [39]. After being in use for 1 year, we updated our ERAS protocol and added a recommendation of rapid awakening, whenever assessed safe and possible; the patient should have a guaranteed airway and no need for ventilatory support. The surgeon and the anesthesiologist together must decide whether rapid awakening is advisable. The adherence to this recommendation has not been optimal at our institution but is gradually improving. It might be that the patients are easier to manage in the ICU during the night following operation when the sedation is continued until the next morning. In addition, old unproved beliefs that prolonged immobility somehow protects the microvascular anastomosis may affect these practices. Many studies have shown that these patients benefit from rapid awakening [17, 18, 27]. According to the study by Clemens et al. the patients that benefit most from this are older HNC patients and those with significant comorbidities with limited cardiopulmonary reserve [39].

In the current study, the use of the ERAS protocol had no effect on mortality within 30 days or 6 months after surgery. In a recently published systematic review and meta-analysis by Chorath et al., 4 of the 16 studies included data on early mortality and there was no difference between patients treated according to the ERAS guidelines and those that were not [6].

The strength of our study is the reasonably large number of patients compared to many other similar studies. There are some limitations that should be considered when interpreting our data. This study has limitations related to possible biases caused by the retrospective design. The patient groups are heterogeneous with possible confounding factors. For reasons unclear to us, the UICC cancer stages of the patients were not evenly distributed: in the pre-ERAS group there were more patients with stage IV disease than in the ERAS group. We hypothesize, however, that this does not cause a remarkable bias, because the same kind of surgery with microvascular flap reconstruction was performed in all patients. In addition, we did not monitor the adherence to the ERAS protocol during the study period. Only in a few studies, has the adherence to the ERAS protocol been monitored in HNC patients [12, 15, 20]. The ERAS protocol includes multiple interventions which makes it difficult to demonstrate the benefits of a single intervention.

## Conclusion

In conclusion, the implementation of the ERAS protocol for major HNC surgery at our institution was feasible and safe and resulted in shortening of LOS, ICU–LOS, and reduced rate of medical complications, while no effect was found on the rate of surgical complications or short-term postoperative mortality. The multimodal nature of the ERAS protocol warrants updating and collaboration between different disciplines to improve its application.

### Supplementary Information

Below is the link to the electronic supplementary material.

Supplementary file1 (XLSX 10 KB)

Supplementary file2 (XLSX 12 KB)
