# Correction: Enhanced recovery after surgery (ERAS) protocol improves patient outcomes in free flap surgery for head and neck cancer

**DOI:** 10.1007/s00405-024-08520-4

**Published:** 2024-03-01

**Authors:** Teija Nieminen, Laura Tapiovaara, Leif Bäck, Andrew Lindford, Patrik Lassus, Lasse Lehtonen, Antti Mäkitie, Harri Keski-Säntti

**Affiliations:** 1 Department of Perioperative and Intensive Care Medicine, University of Helsinki and Helsinki University Hospital, Haartmaninkatu 4, PO Box 340, 00029 HUS Helsinki, Finland; 2 Department of Otorhinolaryngology, Head and Neck Surgery, University of Helsinki and Helsinki University Hospital, Helsinki, Finland; 3 Department of Plastic Surgery, University of Helsinki and Helsinki University Hospital, Helsinki, Finland; 4 HUS Diagnostic Center, University of Helsinki and Helsinki University Hospital, Helsinki, Finland; 5 Research Program in Systems Oncology, Faculty of Medicine, University of Helsinki, Helsinki, Finland

**Correction: European Archives of Oto-Rhino-Laryngology (2024) 281:907–914** 10.1007/s00405-023-08292-3

In the original publication of the article, under the section “Discussion”, the beginning sentence of third paragraph was published incorrectly as “The flap loss rate in the ERAS group was surprisingly high (Tables 3, 4).” The correct sentence should read as “The flap loss rate in the ERAS group was surprisingly high (Table 3).”

In addition, under the section “Discussion”, the following sentence “Instead, the rate of surgical site infections and pneumonias decreased, and the changes were statistically significant in the modified Poisson regression model (Table 4).” was incorrect. The correct sentence should read as “Instead, the rate of surgical site infections and pneumonias decreased, but the changes were not statistically significant in the modified Poisson regression model (Tables 3, 4).”

Similarly, under the section “Discussion”, the following sentence “The rate of re-admissions to the ICU or prolonged stay in the ICU was significantly higher in the ERAS group compared to the pre-ERAS group (Table 4).” was published incorrectly. The correct sentence should read as follows “The rate of re-admission to the ICU or prolonged stay in the ICU was significantly higher in the ERAS group compared to the pre-ERAS group (Table 3)”.

Finally, the statistically significant parameter in Table 4 is only the ‘medical complications in Model 1 and 2 and 3’, all other asterisks indicating statistical significance should be removed. A corrected version of Table 4 is provided.

**Table 4:** Modified Poisson regression model for independent variables comparing ERAS group to pre-ERAS group

Independent variable	Model 1 (variable: ERAS)	Model 2 (variables: ERAS, age, sex)	Model 3 (variables: ERAS, age, sex, stage, ACE-27 score)
	RR (95% CI)	RR (95% CI)	RR (95% CI)
Overall complications	0.96 (0.83–1.15)	0.97 (0.82–1.15)	0.98 (0.83–1.17)
Surgical complications	0.99 (0.78–1.27)	1.00 (0.78–1.28)	1.01 (0.78–1.33)
Medical complications	0.64 (0.46–0.89)*	0.62 (0.45–0.86)*	0.66 (0.47–0.93)*
Type of surgical complication			
Total flap loss	3.19 (1.25–8.15)	3.23 (1.28–8.13)	3.90 (1.42–10.71)
Surgical site hematoma	0.32 (0.09–1.07)	0.32 (0.10–1.09)	0.27 (0.06–1.27)
Surgical site infection	0.61 (0.22–1.70)	0.64 (0.24–1.72)	0.54 (0.14–2.07)
Type of medical complication			
Pneumonia	0.64 (0.25–1.62)	0.61 (0.24–1.57)	0.66 (0.23–1.84)
Delirium	0.60 (0.33–1.10)	0.58 (0.32–1.05)	0.62 (0.33–1.14)
Re-operation within 30 days	0.81 (0.58–1.14)	0.81 (0.58–1.14)	0.90 (0.63–1.28)
Re-admission to ICU/prolonged stay in ICU	1.99 (1.35–2.95)	1.94 (1.32–2.86)	2.02 (1.30–3.15)
Died within 30 days	2.47 (0.60–10.14)	2.27 (0.56–9.23)	1.77 (0.31–10.13)
Died within 6 months	0.99 (0.53–1.86)	0.99 (0.52–1.89)	0.74 (0.30–1.82)

*RR* relative risk, *CI* confidence interval, *ACE-27 score* Adult Comorbidity Evaluation 27, *ICU* intensive care unit

*Statistically significant

